# Supplementary figures and images for: One is not enough: On the effects of reference genome for the mapping and subsequent analyses of short-reads
Source: PLoS Comput Biol. 2021 Jan 27;17(1):e1008678. doi: 10.1371/journal.pcbi.1008678 (PMC7870062; doi:10.1371/journal.pcbi.1008678)

A

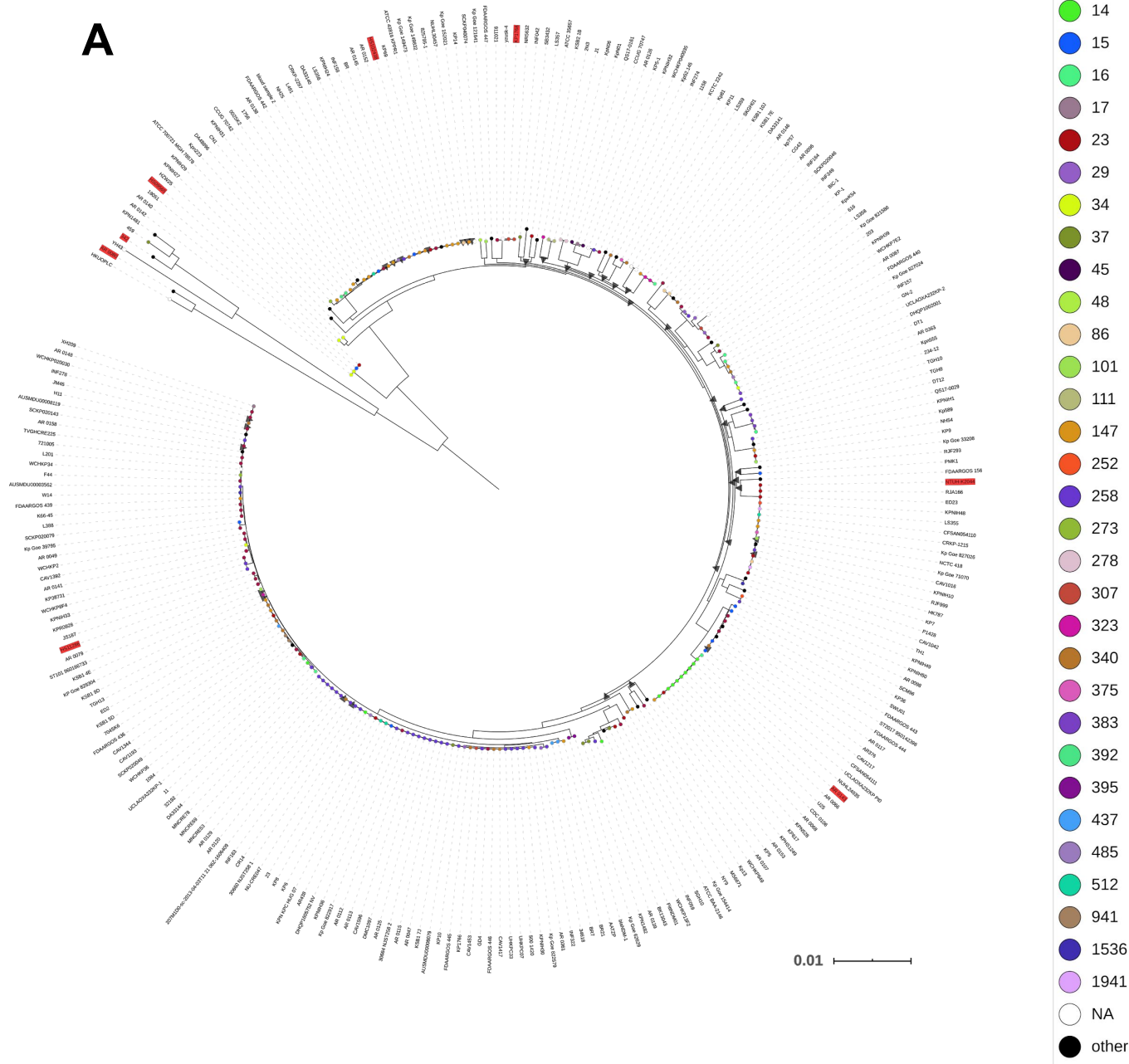

B

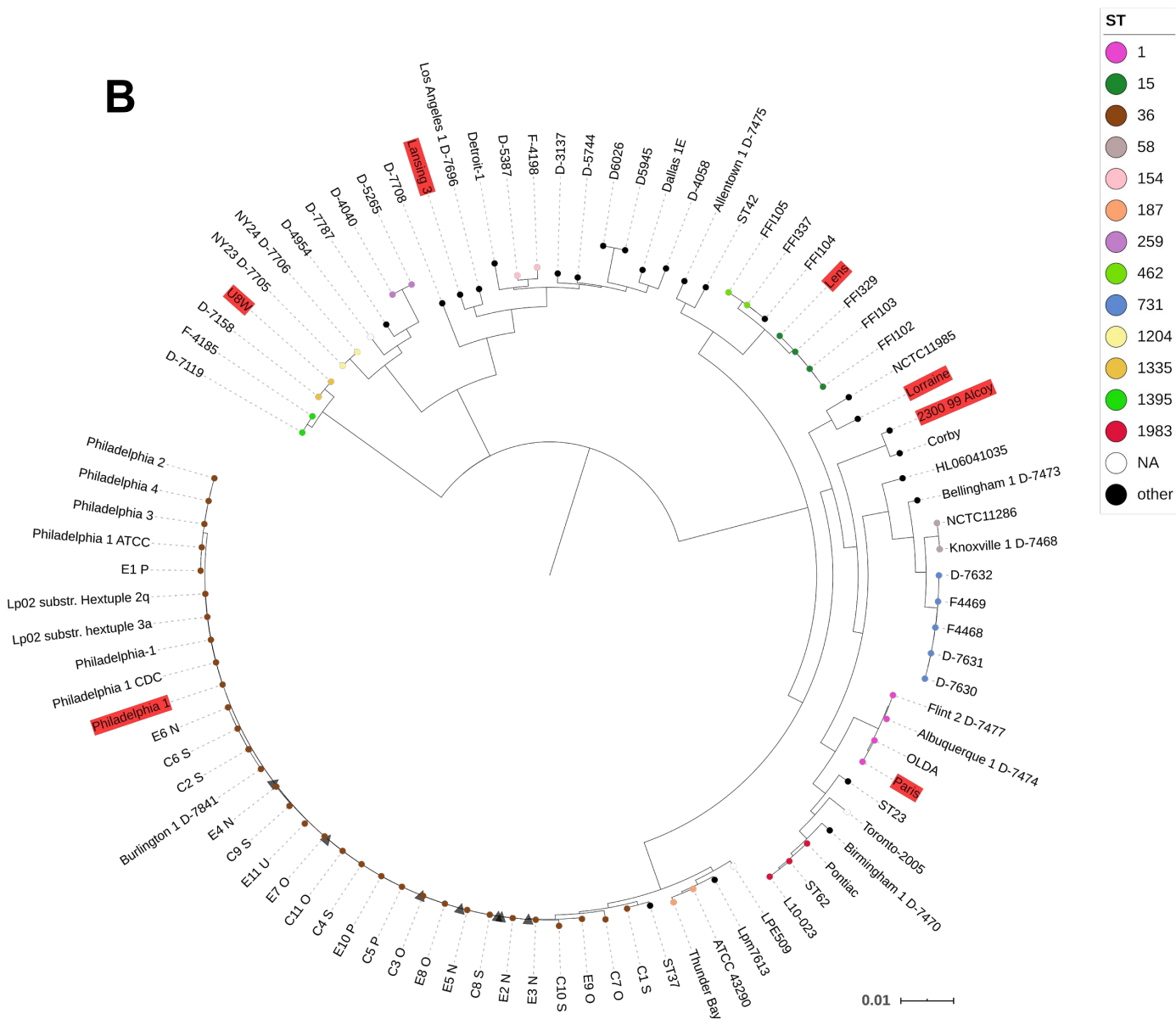

# C

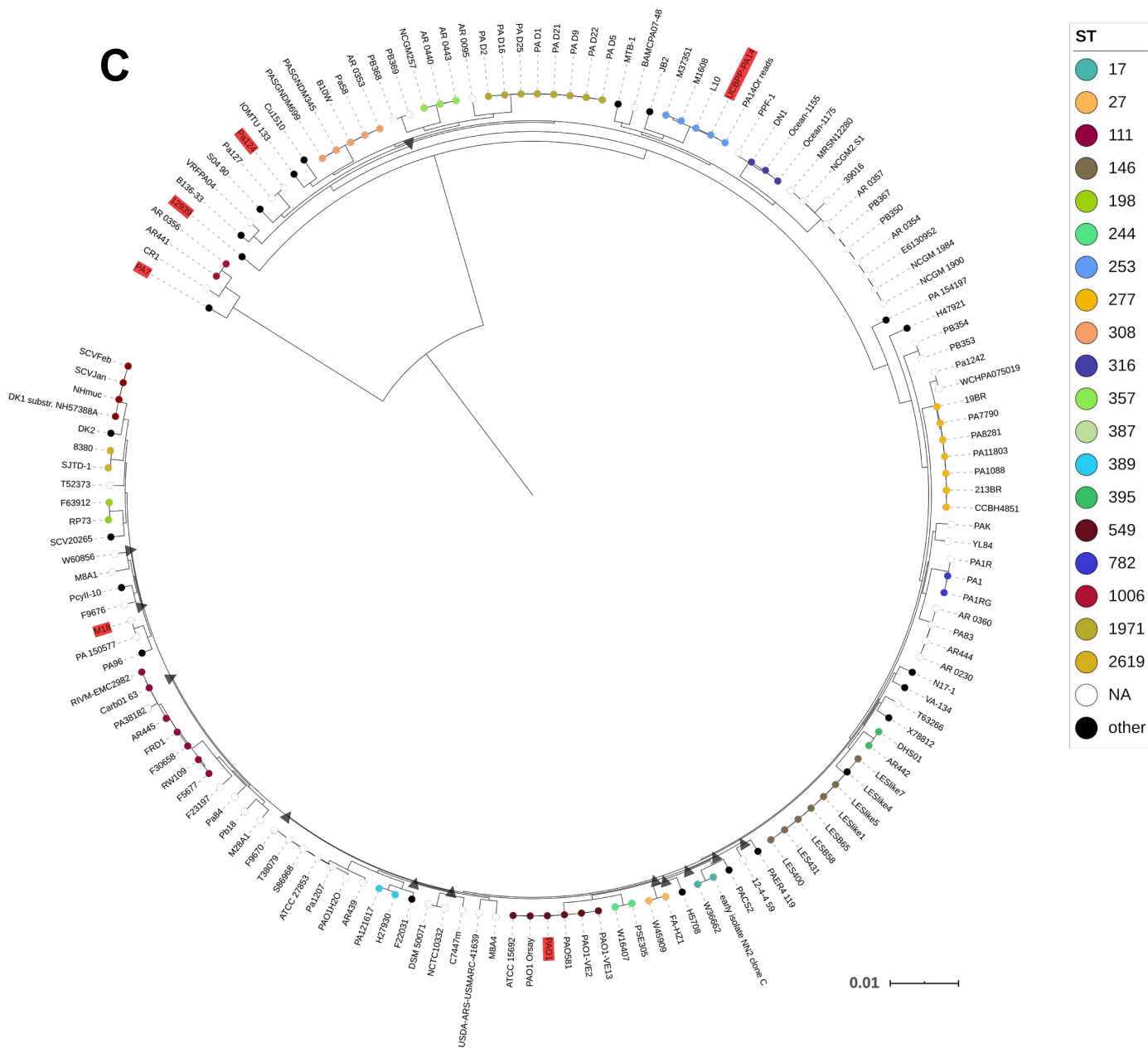

**D**

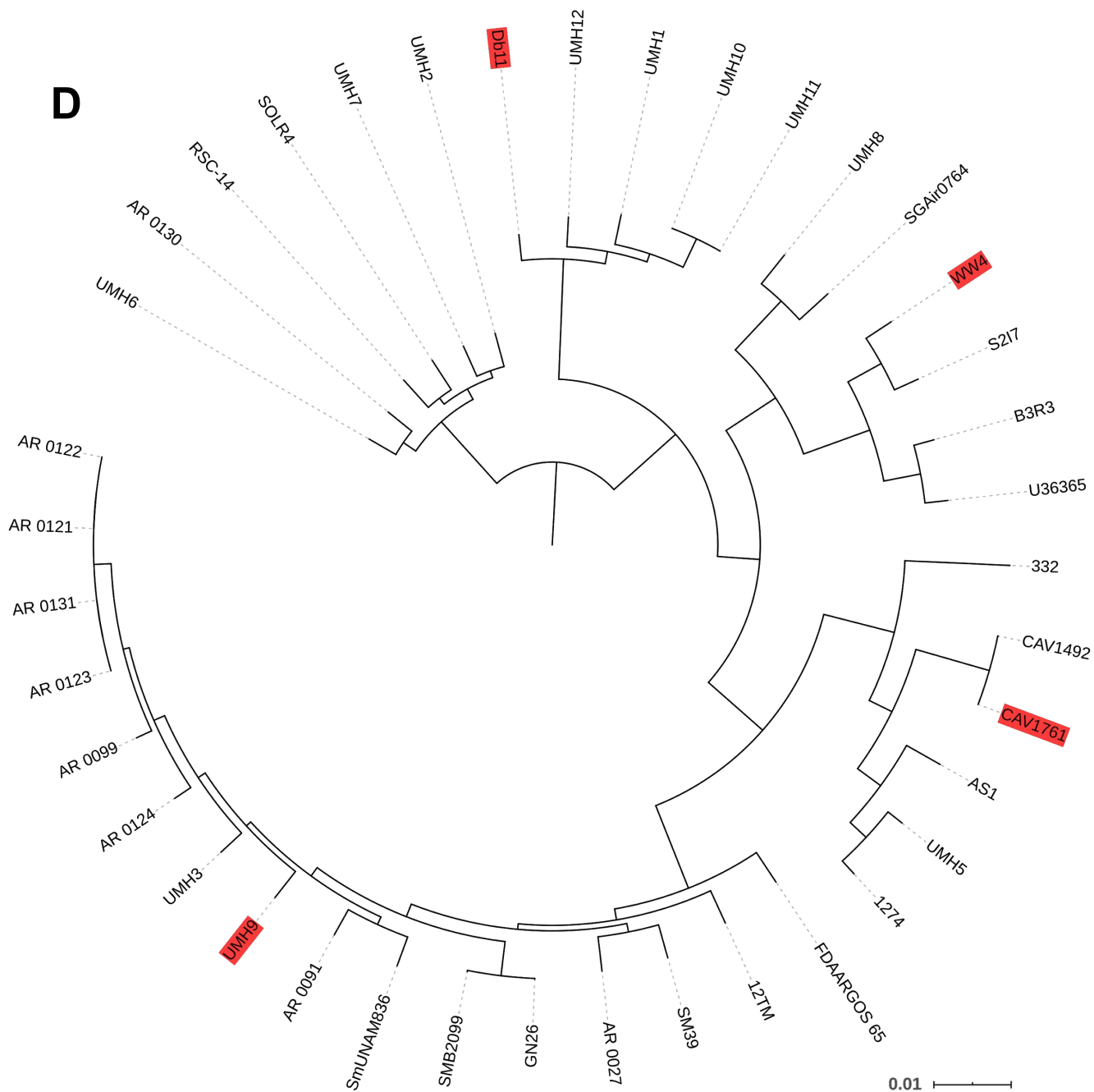

E

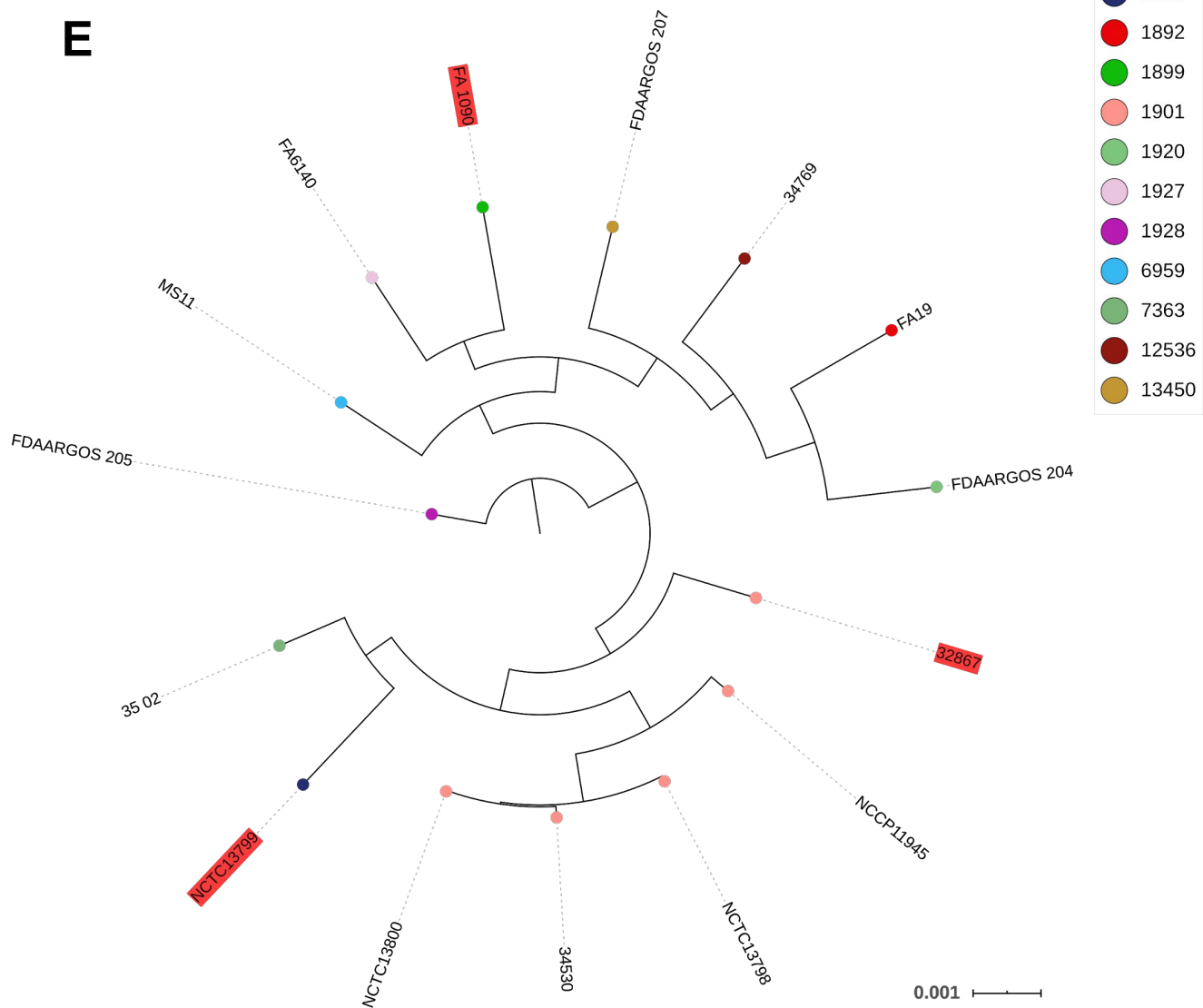

Supplement: S1 Fig — The circles at the tips denote the sequence type (ST) of the different strains in the trees of the species with an MLST scheme available for in-silico typing. The black triangles denote the branches with bootstrap support values <70. (A) K. pneumoniae, (B) L. pneumophila and (C) P. aeruginosa trees were rooted on their corresponding longest branches. As all the branches connecting the different clades of (D) S. marcescens and (E) N. gonorrhoeae trees were approximately the equal length, they were rooted arbitrarily for a better visualization. (PDF) [file pcbi.1008678.s001.pdf]
