# Supplementary material for: One is not enough: On the effects of reference genome for the mapping and subsequent analyses of short-reads
Source: PLoS Comput Biol. 2021 Jan 27;17(1):e1008678. doi: 10.1371/journal.pcbi.1008678 (PMC7870062; doi:10.1371/journal.pcbi.1008678)
Supplement: Attachment — Submitted filename: PLOS-COMP_RESPUESTA_REVISORES.docx [file pcbi.1008678.s011.docx]

**Reviewer #1**: This paper develops authors points out that the choice of a reference in microbial genomics analysis may represent some errors in the detection of SNPs and phylogenetic inference. The procedures are described clearly, and the evidences are convincing.

Comments:

1, I think the Fig. 13 is a very good summary of this paper. To let readers more easily to understand the whole paper, I suggest authors to refer to this figure in Introduction section and rename this as Fig. 1.

2, The code in Github does not include a description on how to use it. The code should contain a detailed manual to make it easier for users to use it.

We thank reviewer 1 for the positive comments on our manuscript.

Comment1: We agree with the reviewer's comment and we have modified the position of Fig. 13.

Comment2: In order to enable the reproducibility of our analyses and to facilitate similar studies by other researchers, we have modified the GitHub code and added a full description of the scripts involved and a usage. Now, there is a single script that can be executed in order to repeat all the analyses performed in this work. After installation of the dependencies, which are detailed and easy to set, the main script can be applied to explore the effects of different references in a custom user dataset (see https://github.com/cvmullor/reference).

**Reviewer #2:** This manuscript systematically analyzed the effects of reference selection in short-reads data analysis, especially its impact in phylogeny reconstruction and epidemiological inferences. I have two main concerns:

1. The novelty of this manuscript is rather limited, as similar work has been done to analyze the effects of reference selection in SNP calling, etc.

We agree with the reviewer that there are previous works analysing the effects of reference selection. However, most of these are focused on the number and quality of SNPs called (see references 23,24,56) and/or only a couple of references are used (see references 27 & 28). Considerably less attention has been given to comparing this effect in the downstream steps of the analysis (phylogenetic inference, natural selection or recombination). There are only a few studies that assess the effects of the reference on phylogenetic trees. For instance, Lee et al., 2016 (see reference 25) used 162 closely related strains of *M.tuberculosis* and 7 different reference genomes to perform a qualitative comparison among the inferred trees. Usongo et al., 2018 (see reference 26) investigated the impact of the reference to distinguish [strains of *Salmonella enterica*](http://paperpile.com/b/SJLgqJ/NgF7F) based on the core genome SNV phylogenetic clustering. To our knowledge, this is the first work to systematically examine the effect of different references for mapping on the inference of tree topology as well as the impact on recombination and natural selection inferences. For example, Walter et al. 2020 (see reference 56) pointed as a limitation of their study that they had not measured the effect of recombination with respect to a reference. Also, we have evaluated the possible consequences for epidemiological analyses, identifying specific changes in the phylogenetic trees that can affect outbreak delimitations for the five bacterial species. The novelty of this work also relies on a procedure that guarantees that we are evaluating only the effect of the reference (see Fig. 1). We assessed that with species of different clonality and differentiating when considering only core or the pangenome. We believe that these differentiating factors make this work highly valuable for researchers using this methodology despite reference choice is a well-known concern.

2, There is no practical guidance on how to select references so that the resulting biases can be reduced. This limits the impact of this manuscripts in practical data analysis.

Despite the merits of this manuscript in quantitatively measuring the effects of reference selection, I wouldn't suggest accepting it mainly due to above two limitations.

The scope of this work is not to provide a solution on the reference selection biases but to provide a general evaluation of how deeply reference choice affects subsequent analyses. This is a known concern but the use of a single reference is still widely used (see for instance references 6, 17, and <https://doi.org/10.1016/j.ijantimicag.2020.106026>). However, as we have shown here this effect is systematic in all species analysed - and these include from low to highly variable representatives - even if we ignore the accessory genome. Also, these effects are not trivial, as phylogenetic inferences can be clearly different. In our concluding remarks, we insist on investigating the effect of different references for each particular case before making any final conclusion. To facilitate this additional analysis, we have made available on the Github page (<https://github.com/cvmullor/reference>) the pipeline used here as a single script. Therefore, users can repeat this analysis for their particular dataset and check whether it is important or not for their practical purposes. There is not an easy way to determine which reference is the best as all the analyses are empirical. Besides, the diversity and uniqueness of each biological dataset impedes to make a generalizable guideline. So, assessing the variance of reference choice is a first step in order to increase conclusions’ robustness. We have added a few lines in the discussion section with recommendations regarding reference selection (see 482-495 or below).

“*Mapping simultaneously to multiple references or against a reference pangenome may alleviate the effect of reference choice. Moreover, when studying particular lineages or outbreaks, using de novo assembled, closely related references (i.e., sequences from the same clade) may also reduce this effect. In any case, exploring the effects of different references on the final conclusions is highly recommended. However, the aim of this work is not to provide a solution on the reference selection bias on mapping but to make clear how deeply reference choice can affect subsequent analyses. Exploring the effects of different references is highly recommended, since it is difficult to unequivocally determine an optimal reference when working with non-simulated reads, further considering that available, high-quality reference genomes may not encompass the complete genomic diversity of a species. Besides, the diversity and uniqueness of each biological dataset impedes the elaboration of a generalizable guideline. Ultimately, inspecting the variability of the results of mapping against different references is an essential step to assess if conclusions are robust to reference choice and which of them are particularly sensitive to the use of specific references.”*
