# Supplementary material for: One is not enough: On the effects of reference genome for the mapping and subsequent analyses of short-reads
Source: PLoS Comput Biol. 2021 Jan 27;17(1):e1008678. doi: 10.1371/journal.pcbi.1008678 (PMC7870062; doi:10.1371/journal.pcbi.1008678)
Supplement: Attachment — Submitted filename: Response_to_reviewers_v2.docx [file pcbi.1008678.s012.docx]

**Comments to the Authors:**

We thank both reviewers for appreciating the revisions made.

Reviewer #1: I have no further comments.

Reviewer #2: In the letter of response to reviewers, the authors made clear the novelty of the work in evaluating the effects of reference selection in recombination, natural selection inferences, and epidemiological analyses, and a new procedure that guarantees only evaluating the effect of the reference. A GitHub repo was well-documented which can help users to evaluate the efforts of the reference with their own data. These addressed my main concerns; I appreciate the authors' efforts.

Two minor concerns / suggestions:

1, I would suggest highlight the *unique* contribution of this work in "Author summary" section, by adding/rephrasing the statements made in the letter, for examples, "To our knowledge, this is the first work to systematically examine the effect of different references for mapping on the inference of tree topology as well as the impact on recombination and natural selection inferences", and "The novelty of this work also relies on a procedure that guarantees that we are evaluating only the effect of the reference".

We have modified the ‘Author summary’ as the reviewer has suggested.

“Mapping consists in the alignment of reads (i.e., DNA fragments) obtained through high-throughput genome sequencing to a previously assembled reference sequence. It is a common practice in genomic studies to use a single reference for mapping, usually the ‘reference genome’ of a species —a high-quality assembly. However, the selection of an optimal reference is hindered by intrinsic intra-species genetic variability, particularly in bacteria. It is known that genetic differences between the reference genome and the read sequences may produce incorrect alignments during mapping. Eventually, these errors could lead to misidentification of variants and biased reconstruction of phylogenetic trees (which reflect ancestry between different bacterial lineages). **To our knowledge, this is the first work to systematically examine the effect of different references for mapping on the inference of tree topology as well as the impact on recombination and natural selection inferences. Furthermore, the novelty of this work relies on a procedure that guarantees that we are evaluating only the effect of the reference.** This effect has proved to be pervasive in the five bacterial species that we have studied and, in some cases, alterations in phylogenetic trees could lead to incorrect epidemiological inferences. Hence, the use of different reference genomes may be prescriptive to assess the potential biases of mapping.”

2, Would it be possible to add a working (small) example to the GitHub repo~(for examples, necessary files in references and reads folders)?

We have added a test folder in the GitHub repo (see <https://github.com/cvmullor/reference/tree/master/test>) with a subset of reads and the rest of necessary files to analyse the Serratia dataset.
